# Complete sternal cleft individualized repair using a 3D-printed polyether ether ketone model: a case report

**DOI:** 10.1093/ehjcr/ytad528

**Published:** 2023-10-24

**Authors:** Guangjian Zhang, Lei Wang, Peizhu Dang, Yang Yan

**Affiliations:** Department of Thoracic Surgery, The First Affiliated Hospital of Xi’an Jiaotong University, Xi’an, Shaanxi, P.R. China; Department of Thoracic Surgery, Tangdu Hospital, The Air Force Military Medical University, Xi’an, Shaanxi, P.R. China; Department of Cardiovascular Medicine, The First Affiliated Hospital of Xi’an Jiaotong University, Xi’an, Shaanxi, P.R. China; Department of Cardiovascular Surgery, The First Affiliated Hospital of Xi’an Jiaotong University, Xi’an, Shaanxi, P.R. China

**Keywords:** 3D printing, Polyether ether ketone, Complete sternal cleft repair, Bicuspid aortic valve, Case report

## Abstract

**Background:**

Bicuspid aortic valve (BAV) is a common anatomical variation that the aortic valve possesses two functional cusps. Sternal cleft is a rare congenital malformation which is caused by failed fusion of sternal bones. Early surgical repair is advised; otherwise, alternative surgical techniques should be performed. Due to their biocompatibility and elasticity, 3D-printed polyether ether ketone (PEEK) implants can be used. Complete sternal cleft coexistence with BAV is infrequent.

**Case summary:**

A 49-year-old man with a 6-month history of paroxysmal shortness of breath and exertional chest tightness presented to our hospital. The man was diagnosed with BAV with severe aortic valve regurgitation and a complete sternal cleft. He underwent aortic valve replacement surgery using the bovine pericardial aortic valve. Concurrently, a 3D-printed PEEK implant surgery was performed to address the sternal cleft. The patient’s postoperative recovery was uneventful.

**Discussion:**

In this case, 3D-printed PEEK implants were used for high biocompatibility and elastic modulus. However, because PEEK material inherently lacks biological activity, enhancing this aspect remains a focal point of clinical research.

**Learning points**

- 3D-printed implants are a safe and effective way to repair sternal cleft in patients who have missed the period when they can be repaired by direct closure. 3D-printed implants allow the personalized repair of chest wall defects while completely matching the mechanical properties of cortical bone.
- Titanium prostheses are the most common implant for chest wall reconstruction. Compared with titanium prostheses, polyether ether ketone implant has more advantages in biocompatibility and elastic modulus, avoiding metal allergy and bone resorption resulting from stress shielding.

## Introduction

Sternal cleft, a rare congenital malformation, often coexists with other defects, including pentalogy of Cantrell, tetralogy of Fallot, and gastroschisis.^1,2^ Pentalogy of Cantrell is characterized by cardiac abnormalities, including enlargement and valvular abnormalities, which can coexist with sternal cleft. The neonatal period is the optimal time for sternal cleft repair,^2^ and once this period is passed, alternative surgical procedures should be chosen. Reconstruction methodologies include primary closure, grafting, muscle flap interposition, or prosthetic implantation.^3^ Here, we report a case of complete sternal cleft coexistent with bicuspid aortic valve (BAV) and aortic valve regurgitation (AR), receiving 3D-printed polyether ether ketone (PEEK) implant surgery and aortic valve replacement surgery.

## Summary figure

**Table:** 

Time	Events
0-month	A 49-year-old man presented to our outpatient clinic with paroxysmal shortness of breath (SOB) and exertional chest tightness. Echocardiogram revealed a BAV malformation and AR, with a regurgitation area of 6.7 cm^2^.
6-month	Aggravation of SOB.
6-month + 5-day	Hospitalization and rechecked echocardiogram identified a BAV with severe AR, regurgitation area was 7.0 cm^2^, and reduced left ventricle (LV) function with ejection fraction was 44.0%.
6-month + 16-day	Surgical intervention was performed, including aortic valve replacement using Edwards Inspires bovine pericardial bioprosthetic valve and sternal reconstruction.
12-month	The patient experienced a smooth postoperative recovery, and echocardiography demonstrated the proper functioning of the bioprosthetic aortic valve with no evidence of perivalvular leakage.

## Case summary

**Figure 1:**
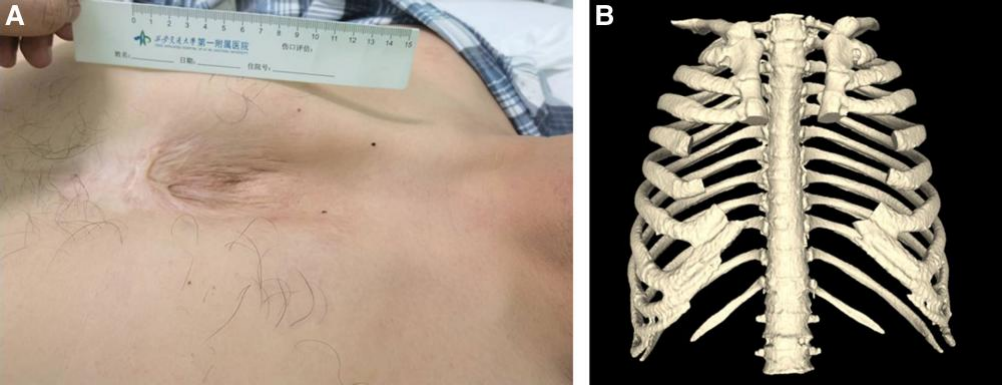
On examination, the midline of the anterior chest wall presented as a sunken V-shaped area with thin skin (*A*). Chest computed tomography 3D reconstruction confirmed a high cardiothoracic ratio and a complete sternal cleft (*B*).

A 49-year-old Asian man with a history of BAV, complete sternal cleft, and hypertension presented with 6-month paroxysmal SOB during activity and exertional chest tightness. Physical examination showed a sunken V-shaped area in midline of the anterior chest wall (*Figure 1A*; see Supplementary material online, *Video S1*). Cardiac examination revealed an enlarged border of cardiac dullness and a diastolic murmur at the aortic area. The echocardiogram from 6 months ago indicated an effective regurgitant orifice area (EROA) of 6.7 cm^2^, with aorta flow velocity of 274 cm/s. The EROA ≥ 0.3 cm^2^ indicates severe AR.^4^ Subsequently, the patient regularly took spironolactone, hydrochlorothiazide, betaloc, and Norvasc but stopped 5 days ago; then, he experienced a worsening of SOB and subsequently came to our hospital.

**Figure 2:**
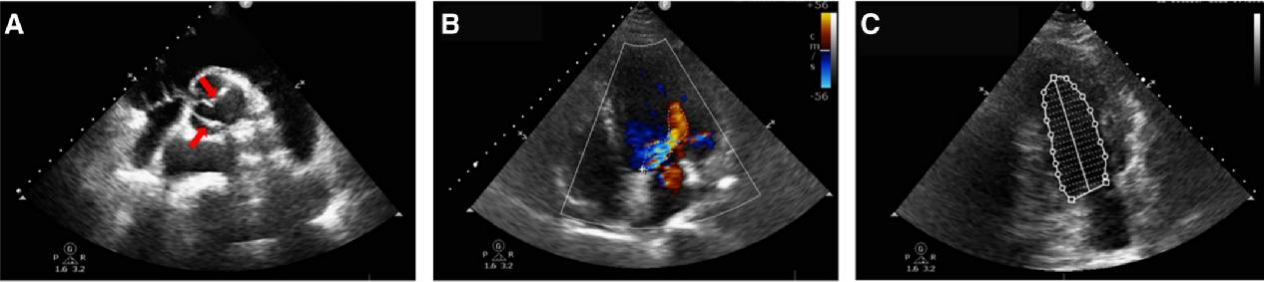
Transthoracic echocardiography showed bicuspid malformation (arrows) (*A*), regurgitation (regurgitation area of 7.0 cm^2^; dotted box) of the aortic valve (*B*), and the systolic dysfunction (biplane Simpson’s method: ejection fraction 44.0%) of the left ventricle (*C*).

Transthoracic echocardiography showed BAV malformation and regurgitation (area: 7.0 cm^2^) (*Figure 2A and B*), a jet velocity of 224 cm/s, and a 20 mmHg gradient. The LV exhibited systolic dysfunction (ejection fraction: 44.0%) (*Figure 2C*) and an end-diastolic diameter of 60 mm. The tricuspid annular plane systolic excursion (TAPSE) measured 21 mm, and the ascending aorta diameter was 49 mm. Chest computed tomography (CT) 3D reconstruction confirmed a high cardiothoracic ratio and a complete sternal cleft (*Figure 1B*). A CT aortogram displayed an ascending aorta aneurysmal dilation and aortic valve calcifications. Other congenital malformations were not found in CT aortogram. Laboratory results highlighted elevated N-terminal prohormone of brain natriuretic peptide at 1160 pg/mL and troponin T at 0.023 ng/mL.

**Figure 3:**
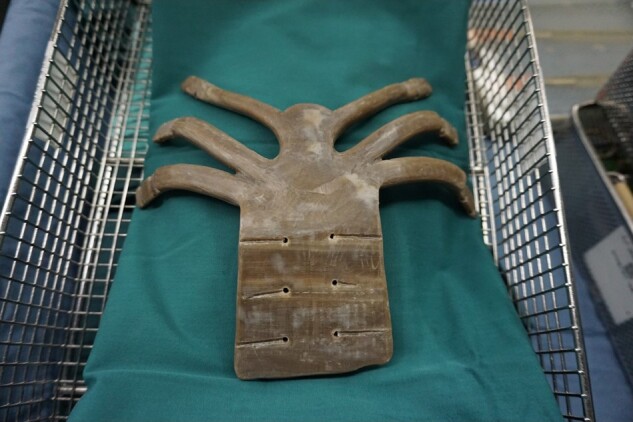
The 3D-printed polyether ether ketone implant.

Due to heart compression risks from direct sternal cleft suturing, a multidisciplinary team proposed surgical reconstruction with a 3D-printed implant. We exported chest CT images in Digital Imaging and Communications in Medicine (DICOM) format and used Geomagic Studio (version 2012; 3D Systems) to perform a smoothing operation for surgical planning. Subsequently, a 3D-printed implant was fabricated with PEEK materials (Victrex, Thornton-Cleveleys) in a fused deposition modelling 3D-printed machine (Jugao-AM-Doctor, Shaanxi Jugao-AM Technology Co, Ltd; melting temperature 340°C). Finally, the implant was processed by ultrasonic cleaning, ethylene oxide sterilization, and disinfection monitoring to satisfy surgical grade (*Figure 3*).

**Figure 4:**
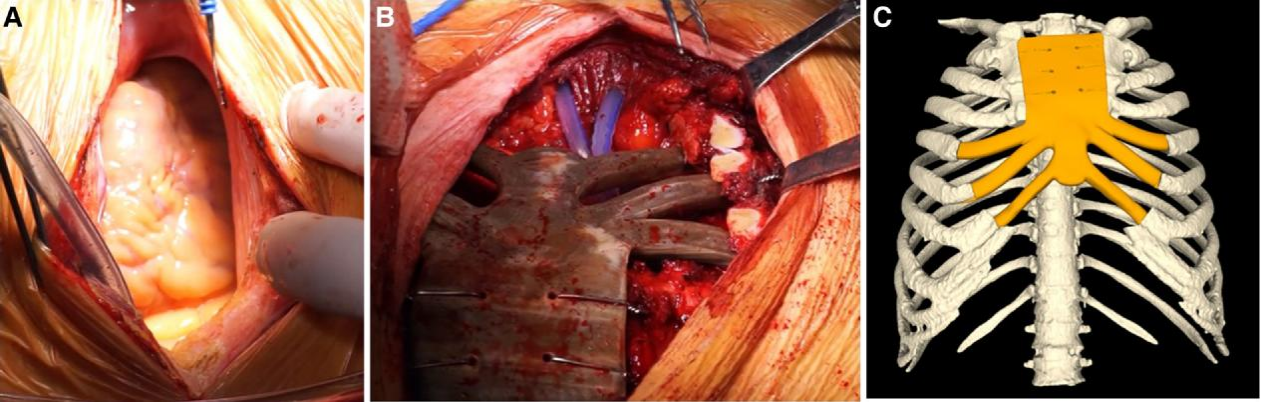
The complete sternal cleft was observed in situ at the time of open-heart surgery (*A*). 3D-printed polyether ether ketone implants to fill bone defects were observed in situ at the time of open-heart surgery (*B*). 3D reconstruction confirmed the polyether ether ketone implant has been placed in the appropriate position (*C*).

A complete sternal cleft was observed in situ during open-heart surgery (*Figure 4A*). First, the patient received aortic valve replacement using an Edwards Inspires NO.25 bovine pericardial valve without aortic root repair or replacement. Postoperative echocardiography confirmed optimal valve function without perivalvular leakage. A foveal deformity was found in the lower part of the sternal cleft. The residual sternum and cartilages 4–7 were excised. The 3D-printed PEEK implant was used to fill the defect (*Figure 4B* and *C*, see Supplementary material online, *Video S2*), and was anchored with steel wires. The bilateral pectoralis major muscle was dissected and the surgical incision sutured.

**Figure 5:**
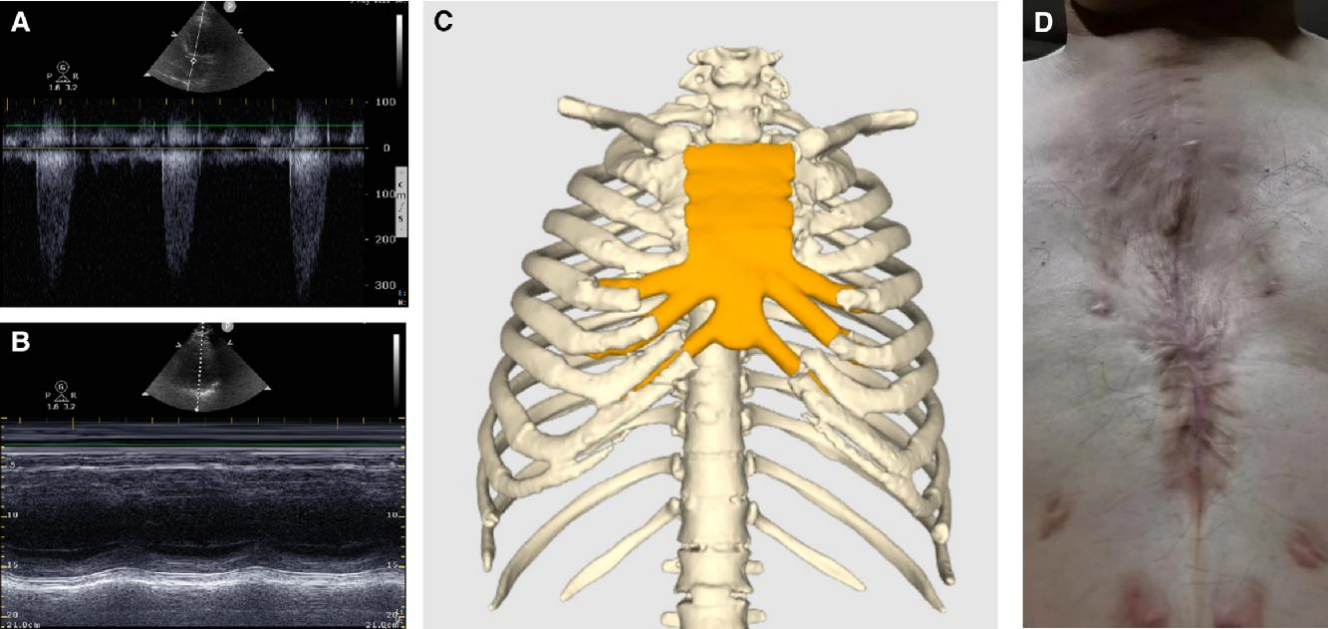
Transthoracic echocardiography revealed that bioprosthetic aortic valve worked properly without perivalvular leakage (*A*), and left ventricular systolic function was improved (M-mode echocardiography: ejection fraction 52.0%; *B*). 3D reconstruction image of the chest demonstrated that the location of the polyether ether ketone implant remains stable (*C*). The gross observation of chest wall structures is shown in *panel D*.

The patient had an uneventful postoperative recovery and was doing well at the 16-month follow-up after surgery. Echocardiography revealed the prosthetic valve performed well (*Figure 5A*) without perivalvular leakage, and LV systolic function was improved (ejection fraction: 52.0%, *Figure 5B*). A 3D chest reconstruction confirmed the PEEK implant’s stability (*Figure 5C*), with chest wall structure presented in *Figure 5D*.

## Discussion

Sternal cleft is a rare congenital malformation, representing only 0.15% of all thoracic malformations. Resulting from failed sternal bone fusion during embryogenesis,^2^ it is often associated with other defects, such as pentalogy of Cantrell, tetralogy of Fallot, aortic coarctation, and gastroschisis. Cardiac abnormalities are the characteristic features of pentalogy of Cantrell, including cardiac enlargement, valvular abnormalities, and so on. In contrast, sternal cleft coexistence with BAV and AR is rare.^3^ Genetic and other factors may play significant roles in above two diseases. In this case, the patient was admitted to the hospital due to severe AR symptoms and underwent aortic valve replacement surgery. Concurrently, a 3D-printed PEEK implant surgery was also performed.

Early sternal reconstruction is the preferred treatment for sternal cleft. It is widely accepted that congenital sternal clefts should be repaired surgically during the neonatal period for the compliance of the thorax is higher and primary direct closure is feasible.^2^ However, after this period, alternative surgical methods are recommended to avoid cardiovascular compression. For this patient, a 49-year-old man, we designed and fabricated a 3D-printed implant to individually fit the irregularly shaped chest wall defect created by the removal of this foveal deformity, thus relieving the pressure on the heart.

As a material with mechanical properties similar to those cortical bones, PEEK was selected to avoid stress shielding. 3D-printed PEEK implants facilitate tailored chest wall repair, mirroring cortical bone’s mechanical properties. In chest wall reconstruction, titanium prostheses are predominant, but their higher density and elasticity modulus may cause damage to adjacent structures or restrict respiratory movement. Conversely, PEEK implants, offering superior biocompatibility and elasticity, mitigate risks of metal allergies and stress shielding–induced bone resorption.^5^ A study on 18 patients with 3D-printed PEEK chest implants reported preserved respiratory function but significantly reduced forced vital capacity over 6–12 months. Postoperatively, our patient exhibited restrictive respiratory dysfunction, potentially due to surgical sequelae.^6^ Lack of bioactivity is a disadvantage of PEEK materials, which may affect bone fusion and cause bacterial infection. Therefore, modifying the surface of PEEK to improve its osseointegration ability and antibacterial activity has been a research hotspot in recent years.^5^ At present, 3D-printed PEEK implants are still in the clinical research stage in our institution; thus, they are provided freely to patients with corresponding surgical indications.

## Conclusion

Sternal cleft, a rare congenital anomaly, can be associated with midline fusion defects. It often necessitates neonatal reconstruction. Beyond this period, tailored interventions, like 3D-printed PEEK implants, become pivotal. Our patient’s successful recovery post PEEK implant and aortic valve replacement underscores PEEK implant potential efficacy in sternal cleft repair.

## Supplementary Material

ytad528_Supplementary_Data

## Data Availability

The data underlying this article are available within the article and in its online Supplementary material.

## Lead author biography

Guangjian Zhang, MD, is the Chief Consultant Surgeon in Thoracic Surgery and Associate Professor of Surgery at Xi’an Jiaotong University (XTJU). He is the Director of the Thoracic Surgery Department at the First Affiliated Hospital of XJTU. Dr Zhang is primarily interested in lung transplantation and complex minimally invasive thoracic surgery including robotic surgery and video-assisted thoracoscopic surgery. He has managed complex poly-trauma cases and chest wall reconstruction using 3D printing. He currently holds the top position in the number of lung transplants in the northwest region of China.
